# Morphological Analysis Reveals a Compartmentalized Duct in the Venom Apparatus of the Wasp Spider (*Argiope bruennichi*)

**DOI:** 10.3390/toxins13040270

**Published:** 2021-04-09

**Authors:** Henrike Schmidtberg, Björn M. von Reumont, Sarah Lemke, Andreas Vilcinskas, Tim Lüddecke

**Affiliations:** 1 Institute for Insect Biotechnology, Justus Liebig University of Gießen, Heinrich-Buff-Ring 26-32, 35392 Gießen, Germany; henrike.schmidtberg@agrar.uni-giessen.de (H.S.); Sarah.Lemke@agrar.uni-giessen.de (S.L.); andreas.vilcinskas@ime.fraunhofer.de (A.V.); 2 LOEWE Centre for Translational Biodiversity Genomics (TBG), Senckenberganlage 25, 60325 Frankfurt, Germany; 3 Department of Bioresources, Fraunhofer Institute for Molecular Biology and Applied Ecology, Ohlebergsweg 12, 35392 Gießen, Germany

**Keywords:** Araneidae, *Argiope bruennichi*, venom glands, venom duct, morphology

## Abstract

Spiders are one of the most successful groups of venomous animals, but surprisingly few species have been examined in sufficient detail to determine the structure of their venom systems. To learn more about the venom system of the family Araneidae (orb-weavers), we selected the wasp spider (*Argiope bruennichi*) and examined the general structure and morphology of the venom apparatus by light microscopy. This revealed morphological features broadly similar to those reported in the small number of other spiders subject to similar investigations. However, detailed evaluation of the venom duct revealed the presence of four structurally distinct compartments. We propose that these subunits facilitate the expression and secretion of venom components, as previously reported for similar substructures in pit vipers and cone snails.

## 1. Introduction

Venoms have convergently evolved more than 100 times in all major animal lineages, where they are employed as an important functional trait [1,2]. The three principal biological functions of venom are predation, defense and competition [3]. However, at least 11 additional functions have since been described, including venom usage for mating, food storage and even communication [1,3]. This functional diversity is inherently linked to the organization of the venom delivery apparatus. For example, the ability to modulate venom composition and to shape venom profiles can be influenced by the possession of centralized vs. decentralized venom systems and the degree of venom gland complexity [1]. A detailed understanding of the morphological organization of venom delivery systems in animals is, therefore, necessary to understand their function and evolution.

Spiders are a group of terrestrial predators which diversified into almost 50,000 species that conquered all continents except Antarctica during the ca. 400 million years since they first emerged [4]. Consequently, spiders abound with a great level of diversity; they all occupy niches as predatory species and prey predominantly on other arthropods, in particular, on insects. The innovation of venom and associated venom delivery systems was a key event in spider evolution. It is, except for very few examples, omnipresent in spiders and used to overpower prey. Considering this omnipresent nature and the great diversity of spiders, they are commonly acknowledged as the most successful venomous order in the animal kingdom [4,5]. Spider venoms are complex mixtures of small molecules, (glyco)proteins and peptides, many of which have been isolated and tested for pharmacological properties [5,6,7,8,9,10]. However, our knowledge of spider venoms is based on a small and non-representative subset of species, while the vast majority of spider diversity has been largely overlooked [11,12]. Even less is known about the morphological organization of most spider venom delivery systems. Although some detailed studies are available, they have focused on a small number of taxa and tend to be published in older literature sources and/or in languages other than English. Research has prioritized the venom systems of potentially dangerous species such as black widows (*Latrodectus* spp.), brown spiders (*Loxosceles* spp.) and wandering spiders (*Phoneutria* spp.) [13,14,15,16,17,18]. More recent studies have considered lynx spiders (Oxyopidae), wolf spiders (*Lycosa* spp.), furrow orb-weavers (*Larinioides* spp.), tarantulas (*Vitalius* spp.) and tube web spiders (*Segestria* spp.) among others [19,20,21,22,23,24,25,26]. The previous works distinguish three principal units into which the venom system of spiders can be divided [13,14,15,16,17,18,19,20,21,22,23,24,25,26]. The first is presented by the two chelicerae, which serve as the injectors of the system. Second, are the venom glands, in which the venom is synthesized and stored. Each chelae is connected to one gland through a duct, which features the third component of a spider venom system. As the glands are responsible for venom production, it is not surprising that morphological studies have focused on these in most cases. They are embedded in muscle fibers and nerves, which probably modulate venom release. From a histological perspective, spider venom glands have the fact that they are rich in secretory vesicles in common. Lastly, they are located at the prosoma, either within the chelicerae in Mygalomorphae and Mesothelae, or they extend into the body cavity, such as in the case of Araneomorphae. Although, previous studies have delivered valuable insights into the architecture of spider venom systems, currently knowledge is based only on a small fraction of spider diversity and a comprehensive understanding of venom delivery systems requires the investigation of additional taxa.

The family Araneidae (orb-weavers) is the third most speciose spider family, with 3058 commonly accepted species [4]. Araneids prey on insects or other arthropods, and deploy complex and conspicuous orb-shaped foraging webs for hunting [27]. These have drawn widespread recognition among the general public, as well as significant interest in research to uncover the natural history and ecology of orb-weavers [27,28,29,30,31]. In particular, the wasp spider (*Argiope bruennichi*) is frequently used as a model to study mating, silk-spinning, predatory behavior [32,33,34,35,36,37,38], and range-expansion [39,40,41,42]. Given the abundance and diversity of araneids [4], surprisingly little is known about their venom systems. The only araneid venoms that have been evaluated at the molecular level are those from *A. bruennichi* [43] and *Araneus ventricosus* [44], but the morphology of the venom systems has not been investigated in detail. To address the limited understanding of araneid venom system architecture, we carried out a comprehensive analysis of the *A. bruennichi* venom system using light microscopy to provide a framework for future studies in other spider species.

## 2. Results

### 2.1. Outer Morphology

**Figure 1:**
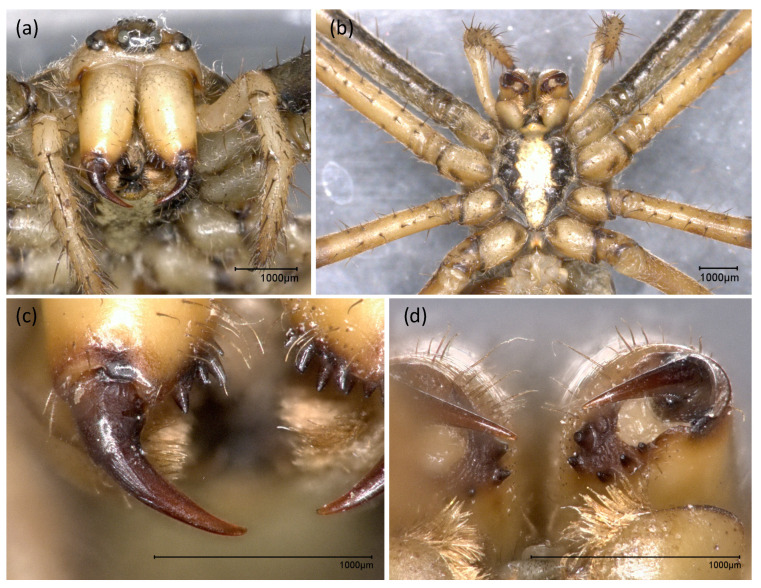
The external morphology of the wasp spider venom apparatus. Anterior (**a**) and ventral view (**b**) on the chelicerae of *A. bruennichi* illustrates the labidognathous orientation of the venom apparatus. It is composed of an enlarged cheliceral basal segment and a small, curved fang at the tip. Magnification of the right fang is given in (**c**). A magnified ventral view on the cheliceral basal segment (**d**) highlights the assembly of cheliceral teeth, forming a pocket into which the fangs can be enfolded.

The wasp spider venom system consists of a pair of chelicerae featuring an enlarged basal segment and a smaller curved fang proximal to the basal segment (Figure 1). The orientation of the chelicerae is labidognathous, which means they are directed towards each other. Some sensory hairs are present on the basal segments. The ventral side of the chelicerae features a series of cheliceral teeth. These form a U-shaped pocket into which the fang is folded in its resting position.

### 2.2. From Fang to Reservoir: Structural Analysis of the Venom Apparatus

The analysis of serial semi-thin sections (referred to as S1–S7) throughout the prosoma illustrated the course and spatial organization of the wasp spider venom system. It starts at the distal part of the paired venom ducts in the cheliceral fangs and ends at the posterior part of the glands within the prosoma.

**Figure 2:**
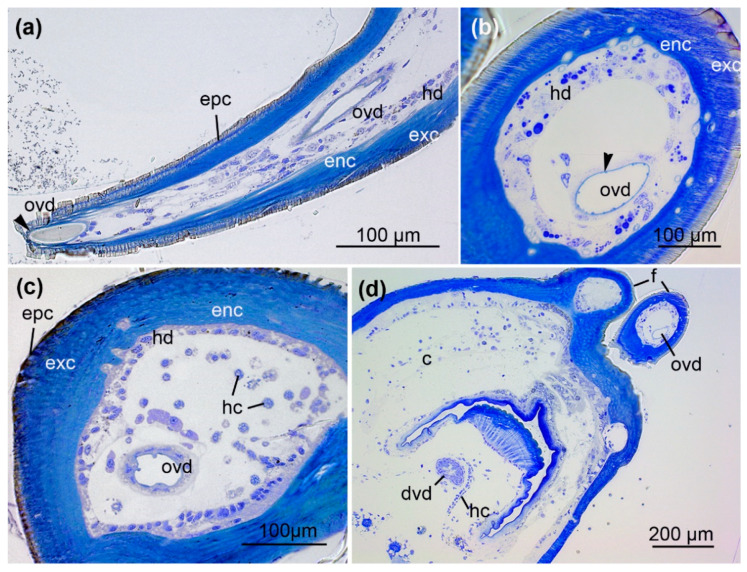
Course of the venom duct in the fang. (**a**) Longitudinal section of a fang near the orifice of the venom duct (arrowhead). (**b**,**c**) Cross-sections of a fang showing the orifical venom duct (ovd). The fang is surrounded by an outer epicuticle (epc), an exocuticle (exc), and inner endocuticle (enc) formed by a hypodermis (hd) consisting of cuboidal epithelial cells. Hemocytes (hc) are also present. The epithelium of the orifical venom duct comprises a thin cuticle layer (arrowhead). (**d**) Cross-section of the chelicera (c) reveals two different venom ducts: the orifical venom duct (ovd) within the fang and the distal venom duct (dvd) in the basal segment. Hemocytes (hc) are arranged along small strands of connective tissue. Sections were stained with 0.5% toluidine blue.

**Figure 3:**
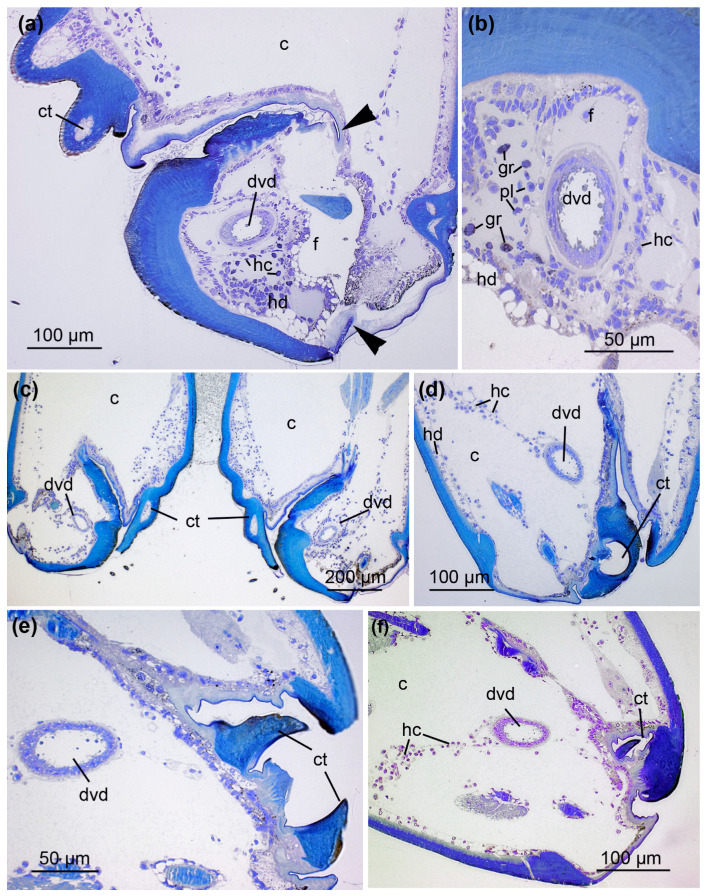
Structure of the chelicera showing the orientation of the distal venom ducts (S2). (**a**) The fang (f) with the distal venom duct (dvd) separates (arrowheads) from the cheliceral basal segment (c). Cheliceral teeth (ct) are present at the ventral surface. Hemocytes (hc) are frequently found in the fang. In some parts of the body, the hypodermis (hd) possesses large vacuoles (see also Supplementary Figure S1). (**b**) Magnification of the distal venom duct (dvd) at the fang base reveals a cuboidal epithelium with a slightly fringed apical region surrounding a broad luminal cavity. Most of the hemocytes (hc) in the fang are plasmatocytes (pm) and, to a lesser content, granulocytes (gr) (see Supplementary Figure S1). (**c**–**f**) Serial sections of a basal segment showing the assembly of cheliceral teeth (ct). Hemocytes (hc) are arranged along small strands of connective tissue. Sections were stained with 0.5% toluidine blue.

According to location and cellular structure, the venom ducts are divided into four discrete sections: (1) the orifical venom duct (ovd) in the distal part of the fangs, which opens with a pore; (2) a distal venom duct (dvd) in the proximal fangs and the cheliceral basal segments; (3) a central venom duct (cvd) in the basal segment; (4) a proximal venom duct (pvd) in immediate contact with the venom gland in the prosoma. All parts show different structural characteristics. The orifice of the venom duct opens near the fang tips (Figure 2a). The fangs are surrounded by a thin epicuticle and an outer exocuticle and inner endocuticle formed by a hypodermis consisting of cuboidal cells (Figure 2b,c). The orifical venom duct in the distal part of the fangs is composed of a flattened epithelium (Figure 2a–c) on a thin layer of connective tissue. Apically, the epithelium comprises a thin cuticle layer (Figure 2b). Because the cellular organization of this orifical duct differs from that of the distal venom duct, we refer to them as two different units. Different hemocyctes, particularly plasmatocytes and granulocytes, are frequently distributed close to the orifical venom duct (Figure 2a,c), and subsequently also to the distal venom duct (Figure 2d and Figure 3).

Subsequent cross-sections of the distal venom duct within the proximal fang and its basal segments reveal cuboidal cells with a slightly fringed apical region surrounding a broad luminal cavity (Figure 3). A few droplet-like granules are distributed over the luminal cavity in this region. The epithelium lies on a thin layer of connective tissue.

**Figure 4:**
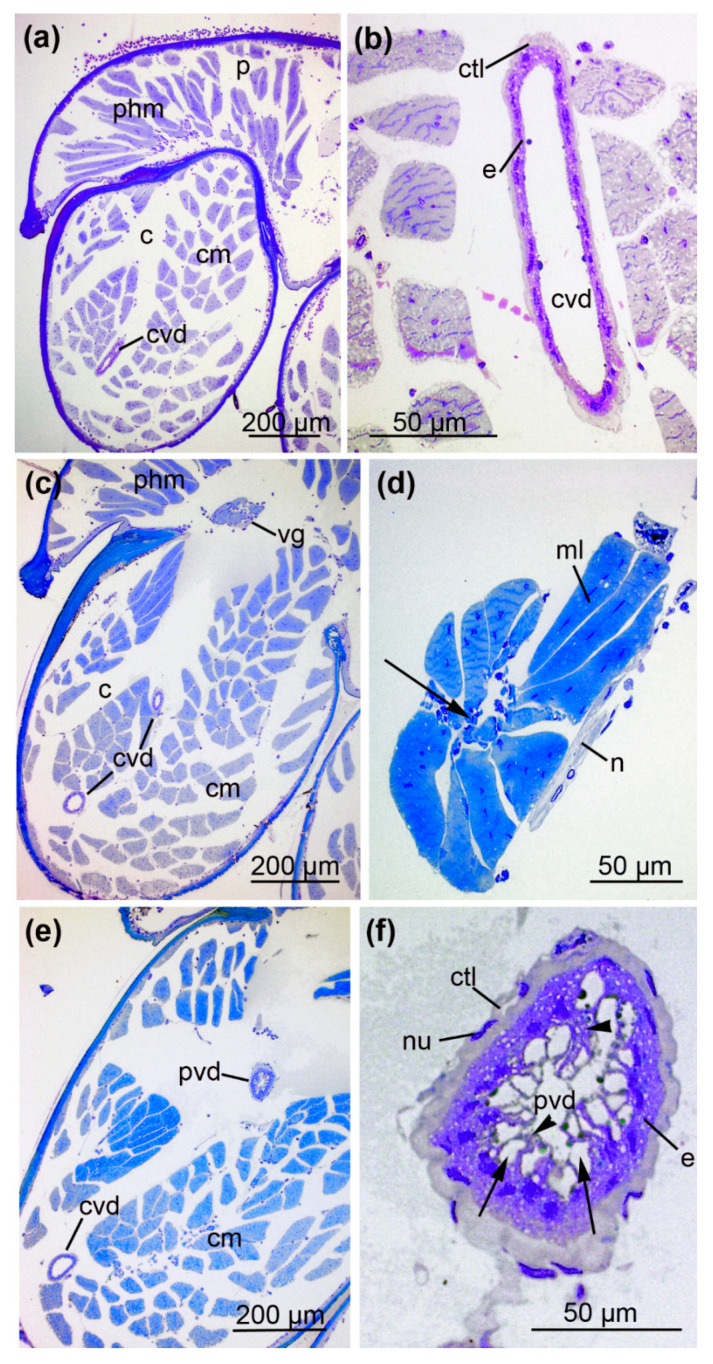
Location of the central and proximal venom duct and the anterior part of the venom gland. (**a**) Cross-section (S3) through the prosoma (p) and cheliceral basal segment (c) including the central venom duct (cvd) plus pharynx (phm) and cheliceral muscles (cm). (**b**) The central venom duct with associated epithelium (e) and surrounding connective tissue layer (ctl). (**c**) Cross-section (S3) illustrating the course of the central venom duct towards the emerging venom gland (vg), which is connected to the pharynx (phm) and cheliceral muscles (cm). (**d**) The anterior part of the venom gland with radially proceeding muscle fibers (ml) enclosing glandular cells (arrow). Nerves (n) are closely connected to the muscle fibers (see also Supplementary Figure S1). (**e**) The proximal venom duct (pvd) can be distinguished from the central duct. (**f**) Magnification of the proximal venom duct (S4). A layer of connective tissue with flattened nuclei (nu) surrounds the epithelial cells. The apical parts of the epithelium build thin projections (arrowheads) that enclose large granules (arrows) towards the lumen. Sections were stained with 0.5% toluidine blue.

The anterior parts of the prosoma and the cheliceral basal segments are packed with muscles inoculating the pharynx in the caudal parts of the prosoma and the chelicera, respectively (Figure 4). At this level, the central venom duct transverses the cheliceral basal segment. It comprises a single layer of flat cells with slender nuclei, supported by a shallow layer of connective tissue (Figure 4b). It proceeds dorsally towards the region of the emerging anterior part of the venom gland (Figure 4c,d).

**Figure 5:**
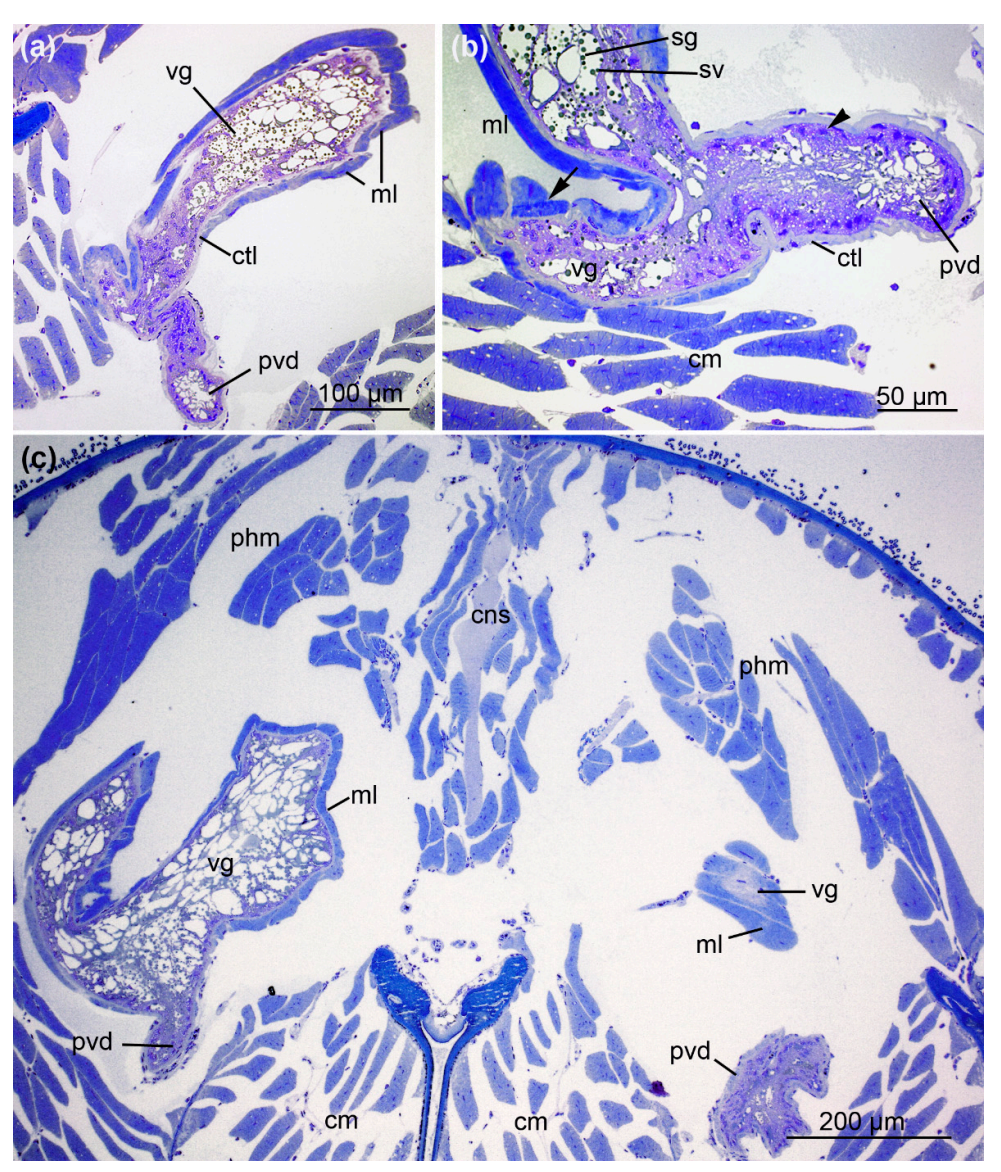
The convergence of the venom duct and venom gland. (**a**) The venom gland (vg), including its secretory granules, is surrounded by muscle fibers. These are connected to the glandular cells via a connective tissue layer (ctl). The proximal venom duct (pvd) reaches the venom gland. (**b**) The proximal venom duct enters the venom gland. The epithelium of the duct shows basally located nuclei (arrowheads) and apical projections, whereas the venom gland epithelium contains secretory granules (sg) and small vesicles (sv). Because of the overlying fibers, the single muscle layer occasionally forms a double layer (arrow) (**c**) Proximal venom duct reaching the anterior part of the venom glands (vg) in the prosoma (cm = chelicera muscles, cns = central nervous system, ml = muscle layer, phm = pharynx muscles, pvd = proximal venom duct). Sections were stained with 0.5% toluidine blue. The images correspond to sections S4 and S5.

The duct undergoes another structural change close to the venom gland, forming the fourth section, referred to as the proximal venom duct (Figure 4e). Here, a thick layer of connective tissue with flattened nuclei surrounds the single layer of columnar epithelial cells. Their apical parts form thin projections that enclose large granules towards the lumen (Figure 4f). The proximal duct ventrally joins the anterior part of the venom gland in the prosoma (Figure 5). This transition is marked by changes in cellular organization. The epithelium of the proximal venom duct features basal nuclei and apical, fringed projections. The epithelial cells of the venom gland are loaded with secretory granules of different sizes as well as small vesicles that become more abundant toward the lumen. In contrast to the venom gland, the proximal venom duct lacks surrounding muscle tissue.

### 2.3. Architecture of the Venom Glands

**Figure 6:**
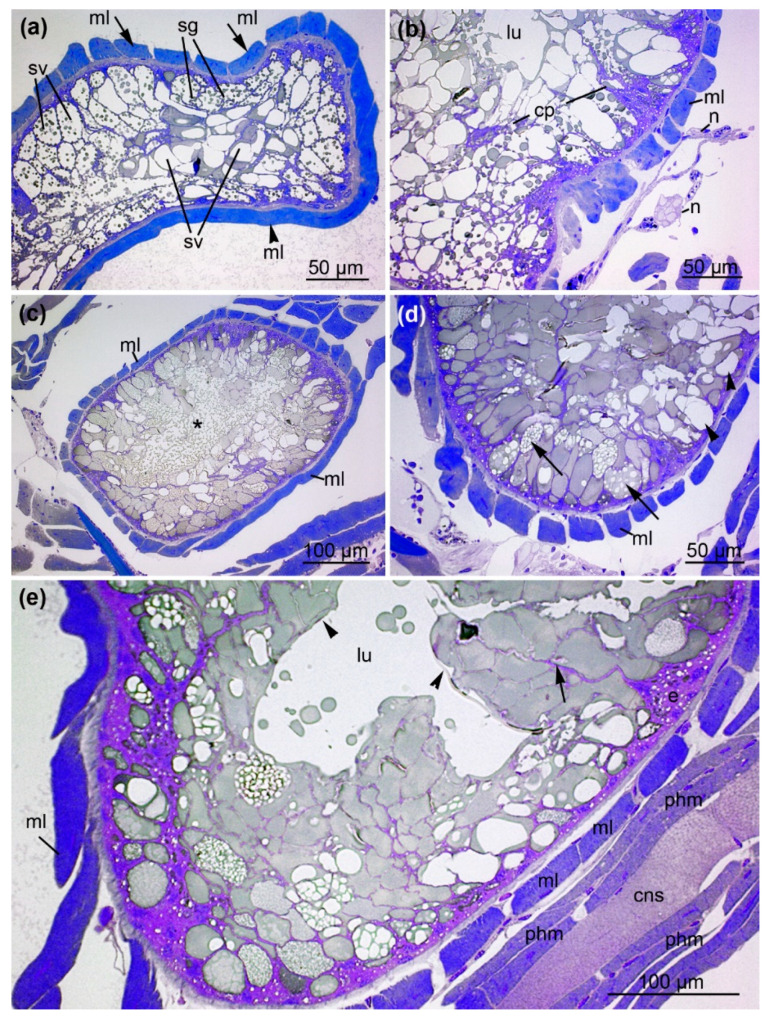
The anterior and central part of the venom gland. (**a**) Cross-section S5 illustrates the anterior part of the venom gland with longitudinally (arrowhead) and transversally (arrow) proceeding muscle fibers (ml). The gland contains secretory vesicles (sv) that are either filled with or lack granules (sg). (**b**) Magnification reveals nerve fibers (n) in close contact with the muscle layer (ml), epithelial cells developing cytoplasmic projections (cp) and luminal cavity (ml). (**c**) Cross-section S6 illustrates the central part of the venom gland where it is widest, enveloped by the muscle layer (ml). The asterisk indicates the holocrine discharge of vesicles into the lumen. (**d**) Magnification illustrates the diversity of secretory granules within the central venom gland epithelial cells. Arrowheads indicate translucent vesicles, and arrows indicate vesicles filled with opaque granules. (**e**) The central part of the venom gland with an empty luminal cavity (lu). An agglomeration of cells forms cytoplasmic projections that reach into the lumen (arrow). A distinct membrane (arrowhead) covers the epithelium. (cns = central nervous system, ml = muscle layer of the venom gland, phm = pharynx muscles).

The anterior part of the venom gland is characterized by radially proceeding muscle fibers that enclose the first glandular cells (Figure 4d). The muscle fibers are obliquely arranged as single layers alongside the gland and are connected to small nerves. In sections, they are longitudinally or transversally aligned (Figure 5a). Occasionally, overlapping muscle fibers form a double layer (Figure 5b). When the gland enlarges, an abundant spongy network formed by the glandular epithelium is incorporated into a thick layer of densely packed muscles (Figure 6). The epithelial cells lie on a basal membrane that is, again, attached to a thick band of connective tissue. The secretory epithelium of the venom glad forms an extended network of interdigitating cytoplasmic processes that enclose secretory granules containing vesicles of various sizes. The vesicles can be isolated or present as agglomerates inside the large secretory granules. In this area, the lumen of the glands is filled with secretory vesicle lacking smaller granules (Figure 6a). Nerve fibers are located along with the venom gland and penetrate the muscle layer (Figure 6b).

The central part of the venom gland reaches its maximum diameter (Figure 6c) and features the greatest structural diversity of secretory granules, suggesting the content of these granules is also diverse. The quantity and density of small, coarse vesicles within the secretory granules is also variable. In some parts of the gland, the entire apical cellular membrane of the epithelial cells dissolves, and numerous small secretory vesicles are discharged and distributed into the lumen. A magnification of this area confirms the diversity of secretory granules from the venom gland epithelial cells (Figure 6d). Most of the secretory granules are densely filled with small opaque vesicles, whereas others appear translucent and empty. As the secretory granules increase in size, the epithelium is divided into units formed by an agglomeration of several cells with cytoplasmic projections reaching into the lumen. Alongside these projections, the large secretory vesicles proceed to the apical parts of the cells.

Towards the posterior prosoma, the central glandular lumen becomes free of granules and vesicles and resembles a canal-like empty luminal cavity (Figure 6e). Distinct cellular membranes of the glandular epithelium enclose large, densely packed secretory vesicles that line the lumen. The sponge-like arrangement of the epithelial apical cell region with its secretory granules, thus, drains into a central compartmentalized lumen. The posterior part of the venom gland is located at the level of the pharynx (Supplementary Figure S2). The diameter of the venom gland decreases towards the posterior end of the prosoma. The central lumen contains large secretory vesicles that yield dense material, whereas the number of secretory vesicles with small granules declines.

## 3. Discussion

### 3.1. Overview of the Wasp Spider Venom System

The wasp spider venom gland is a large organ that reaches deep into the prosoma. It is surrounded by attached muscles, a conformation observed in other spider venom glands. The muscle strands facilitate gland contraction, and thus, control the flow of crude venom. The gland is a complex network of secretory cells that release vesicles to discharge their contents into the lumen. These vesicles are apparently filled with the venom components that are synthesized in the secretory cells. Within these vesicles, the venom components migrate towards the lumen, where the venom is stored until required.

Spiders pierce the body wall of other organisms using their chelicerae, which function as hypodermic needles, secreting venom from their tips [45]. Chelicerates have evolved a variety of different chelae types that differ in their biomechanical properties [46]. The chelicerae of spiders comprise two mobile units—a sharp fang on the tip and a larger basal segment that connects the fang to the prosoma—and thus, function according to the jack-knife principle. In the resting position, the fang is folded against the basal segment, and unfolding allows the spider to inflict a venomous bite, during which venom is released from a small opening close to the tip. Spiders have evolved two different types of chelicerae that can be distinguished by their orientation along the prosoma [46]. In orthognathous chelicerae, both fangs face downwards and work in parallel. In labidognathous chelicerae, the fangs face each other and work similarly to tweezers. Orthognathous chelicerae feature a large basal segment that contains the venom gland and connects the fang to the prosoma [45]. In contrast, labidognathous systems feature a venom gland positioned within the prosoma, and the basal segment is reduced in comparison to orthognaths [45]. Whereas orthognathous systems are found in the two ancestral infraorders Mesothelae and Mygalomorphae, labidognathous systems are present in the Araneomorphae [45]. The evolution of labidognathous chelicerae and the migration of venom glands into the prosoma are thought to have enabled the reduction in body size observed in the Araneomorphae and contributed to its success by allowing the evolution of a web-based lifestyle [6,47].

The wasp spider carries ventral cheliceral teeth that accommodate the fang when the venom delivery system is in its resting position. Cheliceral teeth are found in several spider lineages and enable a secure grip on prey [48]. They are also used to mechanically break up food by species that practice extraintestinal digestion, promoting liquefaction by digestive fluids [45]. Although the wasp spider practices mainly silk-based hunting behavior with limited use of the venom apparatus [34], the presence of cheliceral teeth indicates that the chelicerae are used as versatile tools for handling and consuming prey rather than prey subjugation.

**Figure 7:**
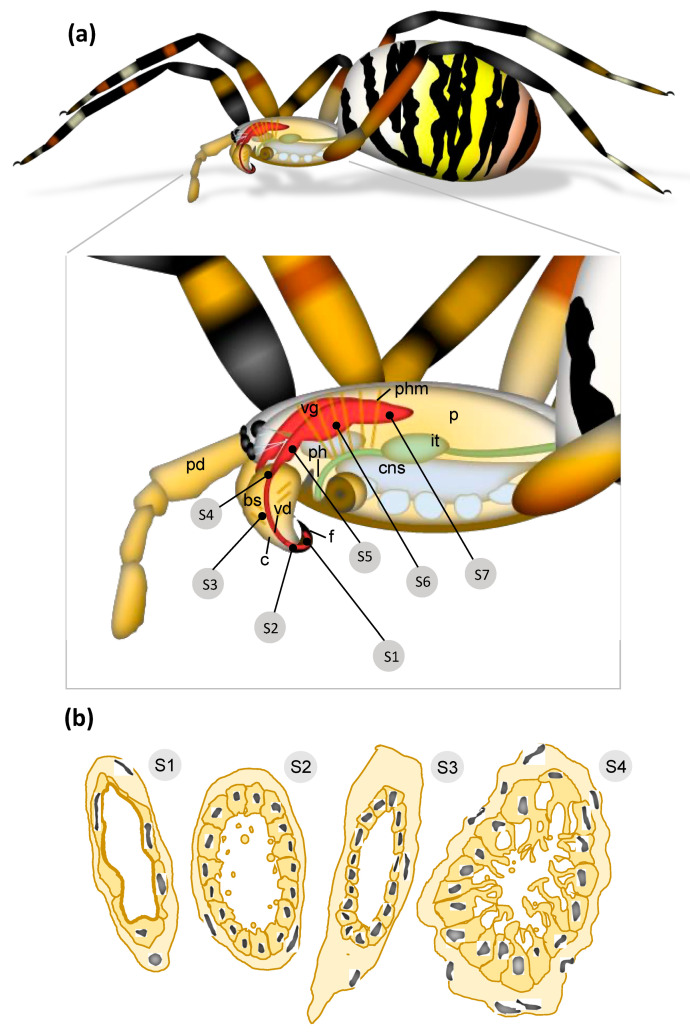
Overview of the *A. bruennichi* venom system. (**a**) The labels S1–S7 indicate the positions of cross-sections discussed in Figure 2, Figure 3, Figure 4, Figure 5 and Figure 6. Within the prosoma lies the central nervous system, an extensive musculature for the extremities and the pharynx, part of the intestinal tract, and a pair of venom glands. (**b**) Schematic drawing of cross-sections representing different subunits of the venom duct. S1: ovd = orifical venom duct of the fang; S2: dvd = distal venom duct of the fang and the distal basal segment; S3: cvd = central venom duct of the basal segment; S4: pvd = proximal venom duct in the basal segment/prosoma. Other abbreviations: bs = basal segment of the chelicera, c = chelicera, cns = central nervous system, f = fang, it = intestinal tract, p = prosoma, pd = pedipalp, ph = pharynx, phm = pharynx muscle, vd = venom duct, vg = venom gland.

In its most proximal sections, the venom duct consists solely of a flat epithelium, forming a thin cuticle layer. However, when the fang proceeds into the basal segment of the chelicerae, it undergoes a range of morphological changes. The diameter of the venom system increases and the basal segment becomes filled with muscle fibers to control the movement of the chelicerae. Externally, the prosoma is rich in pharynx muscles that appear connected to parts of the cheliceral muscle apparatus, suggesting a functional interaction between these systems. This is particularly true for the venom gland, which emerges at the proximal end of the chelicerae. The venom gland is, therefore, embedded in both the cheliceral and pharynx muscles. This arrangement may support the primary muscles along with the venom gland during contraction and envenomation. Supporting evidence includes the connection to small nerves that may regulate and fine-tune the process. The features of the *A. bruennichi* venom system are summarized in Figure 7.

Spiders produce their venoms inside the secretory cells of the venom gland, from which the components are released into the lumen. Essentially, three mechanisms of toxin secretion have been previously proposed for spider venom glands. The first mechanism (merocrine secretion) has sporadically been reported [49]. Here, synthesized proteins migrate via the ER–Golgi apparatus–cell membrane pathway until they are released by exocytosis. The second mechanism (holocrine secretion) is based on protein synthesis at free ribosomes and the release of toxins into the cytoplasm. After several components are synthesized and the cytoplasm is enriched in products, the cell membrane ruptures and releases its contents into the venom gland. Although being a rather destructive mode of secretion, holocrine mechanisms have been reported for several spider venom systems [6,13,14,50]. As it is also encountered in scorpions, holocrine secretion may play a general role in arachnids [51]. Lastly, apocrine secretion, in which parts of the secreting cells form extracellular vesicles, is known as a release mechanism in spider venom glands [6,52]. Based on our data, we suggest a primarily holocrine mechanism of secretion in *A. bruennichi*, facing the degeneration of apical regions in epithelial cells in the central venom gland. Here, the thin cytoplasmic membranes that cover the large apical vacuoles of the epithelial cells are missing. An apocrine secretion, however, is presumed for the distal venom duct in *A. bruennichi* because, here, the epithelium remains intact. However, a precise discussion about releasing mechanisms and the determination of contents has to be continued with further investigations.

### 3.2. Histology of the Venom System

In relation to their diameter, the fangs of *A. bruennichi* are characterized by a comparatively thick cuticle that is composed of an endocuticle, plus an exocuticle that is covered by a distinct epicuticle. A mesocuticle, which is present in some spiders, was not identified here, and its detection presumably requires additional techniques [53,54]. The cuticle of spider fangs displays remarkable physical properties, in that it features a hard and robust structure, but retains a certain degree of flexibility. The exact properties of spider fang cuticle are dependent on several factors, including fibrillary structures and metal incorporation in the different cuticular layers [55]. Overall, the histology of the venom system within the fang seems to support the biting motion in *A. bruennichi*. First, the organisation of cuticles within the fang may add to the overall structural stability during the bite. Second, the cuticle of the fang together with the hemolymph, may act as a powerful counterfort to prevent dilatation of the venom duct when the venom is injected. Vice versa, it may keep the lumen of the small ducts open when not filled with venom. The apically thin cuticular layer of the orifical duct epithelium probably supports this function. Furthermore, its plain surface facilitates a frictionless and fast flow of venom. In comparison, the distal venom duct at the fangs base reveals a cuboidal epithelium with a slightly fringed apical region. Here, vesicles of different sizes are secreted and the epithelium of the proximal venom duct is also characterized by large granules. Both epithelia seem to actively contribute substances to the venom fluid.

Although the position of the gland differs, the morphology of the secretory epithelium of the venom gland of *A. bruennichi* is to a great extent comparable to other described arachnid species: the cell bodies with the nuclei primarily lie in the basal region with apical epithelial processes reaching towards the lumen of the gland [13,14,23]. In *Phoneutria*, Silva and colleagues [14] identified two cell types within the gland epithelium: columnar cells with the nucleus in the apical position, and cuboidal cells with the nucleus near the basal lamina. In *A. bruennichi*, scattered nuclei of epithelium cells are found towards the apical region of the gland epithelium. Due to the large vacuoles, this structure may represent a pseudostratified epithelium. The granules and vesicles in the epithelium vary considerably in size and structure. These alterations are probably caused by varying degrees of cell maturation and aging or by different substances incorporated. The latter may be released and mixed in the gland lumen to the actual venom cocktail.

In several studies of spider venom glands, a thick connective tissue was reported to anchor the gland to two layers of striated muscle fibers [13,14]. In *A. bruennichi*, however, a single layer of muscle fibers encloses the gland. A pseudo-layered appearance is present exclusively at the proximal and distal end of the gland, where the muscle fibers lie adjacently due to the spiral arrangement of the muscle fibers and the decreasing diameter of the gland. The muscles are innervated by small nerve fibers, which lead—after stimulation—to a contraction of the muscles and subsequent squeezing of the venom gland to release the stored venom. The different parts of the venom ducts have no muscle layers, and therefore, the pressure in the gland has to reach the distal parts of the duct. However, two structural features support the flow: (1) their small diameter and (2) the connective tissue layers that surround the ducts. The latter store elastic energy and may help to keep the venom fluid in motion.

Interestingly, we noticed the presence of different hemocytes throughout the venom system of *A. bruennichi*. Such hemocytes perform a variety of cellular reactions such as phagocytosis, encapsulation, and nodulation. Several different hemocyte types are described for spiders, with the most abundant being plasmatocytes and granulocytes. They differentiate from stem cells, the prohemocytes. In particular, granulocytes are have also been suggested to play a role in humoral immune responses by storing immune related compounds [56,57]. Additionally, cyanocytes contain hemocyanin and are, therefore, considered to contribute to respiration [58]. In the wasp spider venom system, plasmatocytes and granulocytes are frequently distributed in the fang and in the basal segment of the chelicera. This may be explained by the need for swift regeneration coupled with immune responses in this area. Fang damage can easily lead to infections, because of the direct contact with prey items and their associated microbiome. Often, the hemocytes are closely assembled to the orifical and distal venom duct. This arrangement is presumably based on small strands of connective tissue that retain the venom ducts in position and thereby lock the hemocytes.

### 3.3. Hidden Complexity of the Venom Duct

Whereas the venom duct is a simple structure in the fang, it gains increasing structural complexity as it proceeds through the chelicerae toward the venom gland. In the chelicerae, it comprises a flattened and simple epithelium with slender nuclei and a layer of connective tissue. The venom duct undergoes further changes proximal to the venom gland. Here, it comprises columnar epithelial cells surrounded by a thick layer of connective tissue and is rich in flattened nuclei. The apical parts of the epithelium form projections enclosing large granules toward the lumen of the duct. These profound differences in cellular organization throughout the venom duct imply four different subunits: an orifical region in the fang, a second distal region and a third central region of the venom duct (extending throughout most of the basal segment in the chelicerae) and a final proximal region close to the convergence of the venom duct and venom gland (Figure 7).

To the best of our knowledge, this type of structural diversification in the venom duct has not been described in spiders before. Few studies have described the role of the venom duct in terms of differential venom secretion, although in cone snails and pit vipers (*Bothrops* spp.) toxins are also expressed by the venom duct and not only by the venom gland [59,60]. Based on our careful analysis, spider venom ducts may be much more important for the functionality of a given venom system than previously understood. First, they form a bottleneck between the venom gland and the fang, which confers the ability to influence injection pressure and hence envenomation efficiency. Second, venom ducts may also fulfill a metabolic and synthetic role in the venom system. The venom ducts of cone snails are divided into distal, central and proximal parts, each of which expresses a different subset of toxins, suggesting evolutionarily specialization for the biosynthesis of specific conotoxins [61]. We found that the wasp spider venom duct is subdivided into four discrete parts, suggesting this structure has also undergone evolutionarily specialization in spiders. The proximal area, in particular, resembles parts of the venom gland, as it contains a loose network of thin projections with large granules. However, in our investigations on *A. bruennichi*, it appears that actively secreting parts (cuboidal to columnar epithelia of the distal and proximal venom ducts) alternate with non-secreting flattened epithelia (orifical and central venom ducts). However, the different epithelia of the duct and the contents of their vesicles and granules have to be verified with further histological and ultrastructural methods. However, a general involvement of the venom ducts in the modulation of the venom fluids should also be considered in spiders.

## 4. Conclusions

The spiders (Araneae) are a highly diverse order of arthropods, in which the overwhelming majority of members utilize a venom system. Research has focused on a small and unrepresentative selection of species, and little is known about the diverse nature of spider venom and even less about the morphology and architecture of the corresponding venom systems. We investigated the venom apparatus of the wasp spider (*A. bruennichi*) and added new information about the largely overlooked family Araneidae. Although we noted superficial similarities with the general morphology and arrangement of other spider venom systems, careful analysis revealed hitherto unknown structural complexity in the venom duct. The venom duct of *A. bruennichi* is divided into four distinct regions: The orifical, distal, central and proximal venom duct. The proximal venom duct, in particular, appears to be involved in a spatially restricted venom function that extends beyond simple secretion. These findings should be explored in more detail and tested using technologies such as MALDI imaging, spatial transcriptomics and 3D high-resolution computer tomography reconstruction.

## 5. Materials and Methods

### 5.1. Preparation of Histological Sections

Adult female wasp spiders were collected during August 2019 from a meadow in Gießen, Germany. The site was previously selected for the collection of wasp spiders for venomic analysis [41]. Captured animals were kept in plastic enclosures (20 × 20 × 20 cm) and were anesthetized and killed with CO_2_ before dissection.

### 5.2. Preparation of Histological Sections

For structural analysis, the prosomata of spiders with both chelicera and their associated venom glands were dissected from the opisthosoma. The tissues were submerged in ice-cold 0.2 M phosphate buffer (pH 7.2) and then fixed with 2.5% glutaraldehyde (Plano GmbH, Germany) in phosphate buffer for 2 h. After washing in phosphate buffer, the samples were post-fixed with 1% OsO_4_ (Roth, Germany) in the same buffer at room temperature for 1 h, followed by washing in tap water, dehydrating through a graded ethanol series, and embedding in Araldite (Plano GmbH, Germany ). Semi-thin sections (1 mm) were prepared using a Leica Reichert Om/U3 ultra-microtome (Leica Microsystems, Wetzlar, Germany). Sections were stained with 0.5% toluidine blue (Roth, Germany) in 0.5% sodium borate (Roth, Germany). The positioning of cross-sections is provided in Figure 7.

### 5.3. Microscopy

The external morphology of wasp spiders was examined by light microscopy on a VHX stereomicroscope (Keyence, Milton Keynes, UK). Histological sections were examined using a DM 4 B microscope (Leica Microsystems).

## Data Availability

Not applicable.

## Supplementary Materials

The following are available online at https://www.mdpi.com/article/10.3390/toxins13040270/s1, Figure S1: Structural details of the hypodermis and the innervation of the venom gland, Figure S2: Posterior part of the venom gland.
