# Supplementary material for: Morphological Analysis Reveals a Compartmentalized Duct in the Venom Apparatus of the Wasp Spider (Argiope bruennichi)
Source: Toxins (Basel). 2021 Apr 9;13(4):270. doi: 10.3390/toxins13040270 (PMC8070055; doi:10.3390/toxins13040270)
Supplement: Supplementary file 1 [file toxins-13-00270-s001.pdf]

Supplementary Materials: Morphological Analysis Reveals a Compartmentalized Duct in the Venom Apparatus of the Wasp Spider (*Argiope bruennichi*)

Henrike Schmidtberg, Björn M. von Reumont, Sarah Lemke, Andreas Vilcinskas and Tim Lüddecke

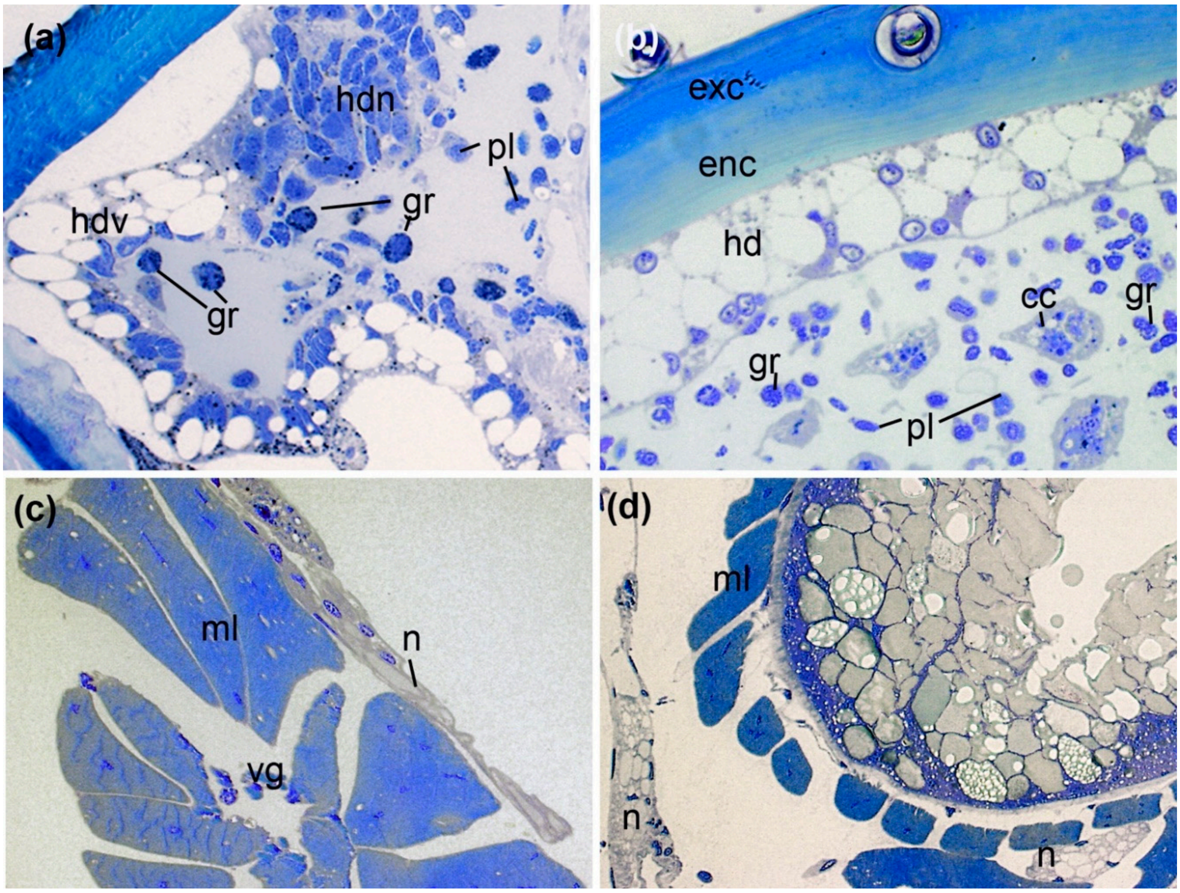

Figure S1. Structural details of the hypodermis and the innervation of the venom gland. (**a**) Magnification of the fang region (see also Figures 2a,b) which is lined by the hypodermis (hd). Here, the hypodermis is artificially detached from the cuticle and different parts of the cells are sectioned. Large vacuoles (hdv) are positioned in the apical part of the hypodermis cells, whereas their nuclei (hdn) are predominately positioned at the cell base. Dark stained granulocytes (gr) can be differentiated from plasmatocytes (pl). (**b**) Large vacuoles in the hypodermis (hd) are found in several parts of the spider's body (here dorsal cuticle with exocuticle (exc) and endocuticle (enc) in the prosoma). Below the hypodermis, numerous hemocytes are found in the hemolymph: granulocytes (gr), plasmatocytes (pl), and cyanocytes (cc) are present in this area. (**c**) Magnification of the same area shown in Figure 4d. Muscle filaments (ml) are surrounding the outermost cells of the venom gland (vg). They are in contact with a small nerve (n). (**d**) Nervous tissue (n) is regularly found in close contact to the venom gland, which is enveloped by a layer of muscle tissue (ml).

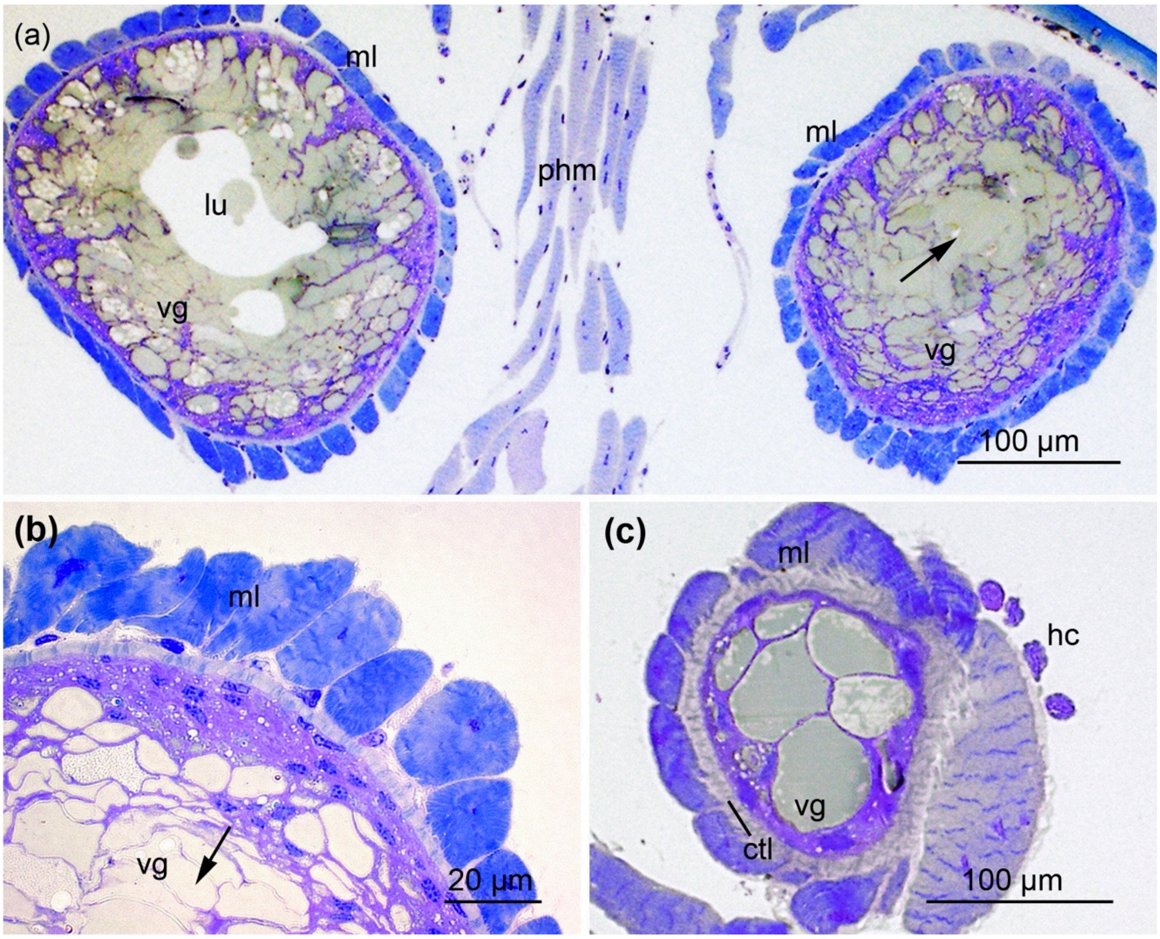

Figure S2. Posterior part of the venom gland. (**a**) Cross-section S7 gives an overview of the posterior part of the venom gland (vg). phm = pharynx muscles. Towards the posterior part of the gland the lumen is completely filled with secretory granules (arrow). (**b**) The magnification reveals secretory granules with differing dense material (arrow). (**c**) Almost posterior end of venom gland. ctl = connective tissue layer, hc = hemocyte, lu = lumen, ml = muscle layer.
